# What are the research priorities for strengthening public health emergency preparedness and response in Africa?

**DOI:** 10.1186/s12961-023-01059-6

**Published:** 2023-10-23

**Authors:** Obinna Onwujekwe, Chinyere Mbachu, Joseph Okeibunor, Godwin Uchenna Ezema, Nonso Ejiofor, Fiona Braka, Adama Thiam, Etien Luc Koua, Dick Chamla, Abdou Salam Gueye

**Affiliations:** 1 Health Policy Research Group, Department of Pharmacology and Therapeutics, College of Medicine, University of Nigeria, Enugu, 400001 Nigeria; 2 Department of Health Administration and Management, College of Medicine, University of Nigeria, Enugu, 400001 Nigeria; 3 Department of Community Medicine, College of Medicine, University of Nigeria, Enugu, 400001 Nigeria; 4 World Health Organisation Regional Office for Africa (AFRO), Brazzaville, Congo; 5 Enugu State Primary Healthcare Development Agency, Enugu, Nigeria

**Keywords:** Emergency preparedness and response, Prioritization, Research priority-setting, African region

## Abstract

**Background:**

Research evidence is needed to strengthen capacities in emergency preparedness and response (EPR). However, the absence of a clear research agenda limits the optimal use of research evidence. This paper reports on the prioritization of research questions and topics that could contribute to evidence-informed strengthening of EPR capacities in the African region.

**Methods:**

The priority-setting consisted of desk review and stakeholder consultation workshop. Twenty-nine people participated in the workshop, including representatives from WHO regional office and EPR focal points in Africa, representatives of research institutions, and partners from Science for Africa Foundation, United Nations Children's Fund and Africa Center for Disease Control. Modified Delphi technique was used to systematically arrive at specific and cross-cutting research priorities in the three broad areas of the EPR, which are program Implementation, Clinical and Epidemiology. The research questions/topics were ranked on five-point Likert scale (1 = very low to 5 = very high) based on seven agreed-on criteria. Research priority score was calculated for each question as the mean of the criteria scores.

**Results:**

A total of 123 research questions comprising, 29 on Epidemiology, 22 on Clinical, 23 on program Implementation, and 49 on cross-cutting issues were ranked. The top ten research priorities were: knowledge and skills of healthcare workers in detecting and responding effectively to disease outbreaks; quality of data (accuracy, timeliness, completeness) for epidemic prone diseases; determinants of vaccine hesitancy; determinants of infection transmission among health care workers during PHE; effective measures for protecting health workers from highly infectious pathogens in PHE; strategies to improve the effectiveness of contact tracing for epidemic prone diseases; effectiveness of current case definitions as screening tools for epidemic and pandemic prone diseases; measures to strengthen national and sub-national laboratory capacity for timely disease confirmation within the Integrated Diseases Surveillance and Response framework; factors affecting prompt data sharing on epidemic-prone diseases; and effective strategies for appropriate community participation in EPR.

**Conclusions:**

The collaborative multi-stakeholder workshop produced a starting list of priority research questions and topics for strengthening EPR capacities in Africa. Action needs to be taken to continuously update the research agenda and support member States to contextualize the research priorities and commission research for timely generation and uptake of evidence.

**Supplementary Information:**

The online version contains supplementary material available at 10.1186/s12961-023-01059-6.

## Background

Globally, there are multiple health emergencies comprising disease outbreaks and humanitarian conflicts and often in challenging settings. The African region especially reports over 100 public health events/emergencies (PHEs) annually [1], of which approximately 80% are emerging and re-emerging infectious diseases, events, and conditions [2]. We define PHE as “any situation whose health consequences have the potential to overwhelm routine capabilities to address them due to the scale, timing or unpredictability of the situation” [3]. PHEs could be of national or international concern depending on geographic spread. Recent public health emergencies in the region include infectious disease outbreaks such as Ebola, COVID-19, and the ongoing Cholera outbreak; man-made disasters such as conflicts and wars in the central African region and the Horn of African; and natural disasters such as cyclones, flooding and drought in the southern African region and the Horn of Africa [4, 5]. These events have significant implications for global health security and universal health coverage gains [6], and are often associated with high morbidity, mortality, and significant socio-economic disruptions.

The impact of unexpected public health events can be significantly minimized if national capacities for emergency preparedness and response (EPR) are strengthened. The International Health Regulations (IHR) stipulates 13 core capacities that are required by countries to be able to effectively detect and respond to public health risks and emergencies. They are: National legislation, policy and financing; Coordination and National Focal Point communications; Surveillance; Response; Preparedness; Risk communication; Human resources; Laboratory; Points of entry; Zoonotic events; Food safety; Chemical events; and Radionuclear emergencies [7]. Although the World Health Organization (WHO) has made significant efforts to facilitate the attainment of the core capacities required under the IHR, many countries in the African region still lack the minimum capacities necessary to predict, plan for, rapidly detect and respond to and recover from public health emergencies.

Evidence from research is needed to understand the drivers of public health emergencies in the African region, and to support the discovery, design and delivery of effective interventions that will contribute to strengthening capacities in EPR [8, 9]. National Health Research Systems are disposed to generate context-specific and relevant knowledge in a timely manner [10]. Entrenching research agenda on health emergencies such as pandemics and stimulating the generation of evidence, translation and dissemination of valuable knowledge will help in enhancing emergency preparedness and response (EPR) to health emergencies. The unpredictability of the events makes it more challenging to rely on old knowledge and systems to contain them. Improving our response to these events requires information and research, this calls for a robust research agenda.

Well-defined health research priorities provide useful guidance in the strategic allocation of research resources, such that the benefits of research investment can be maximized [11]. Although there could be an endless list of research questions that could contribute to strengthening EPR capacities in SSA, the resources to undertake health research are limited. It therefore becomes necessary to identify the research questions that will generate relevant contextual evidence for programme improvements.

Failure to prioritize research questions and topics for ensuring evidence-based decision making will result in the persistence of poor health and weak preparedness against health emergencies in the region. Also, it could lead to misinterpretation of the drivers of poor health, poor articulation of the soundest interventions for strengthening country EPR capacities, and a failure to understand the strategies for optimizing the effectiveness of these interventions [10]. Such failures can accentuate the current state of weak EPR capacities in the region.

This paper reports on the prioritization of research questions and topics that provide the required evidence for evidence-informed strengthening of EPR in sub-Sahara Africa (SSA). It provides new knowledge on the research priorities for strengthening EPR capacities in SSA. It also contributes to existing literature on health system research priority setting.

## Methods

### Study context and design

The WHO AFRO EPR Cluster launched three flagship programmes namely, Promoting Resilience of Systems for Emergencies (PROSE), Transforming African Surveillance Systems (TASS), and Strengthening and Utilizing Response Groups for Emergencies (SURGE) in early 2022. The overarching goal of the programmes is to promote health security in the African Region and contribute to the achievement of the Sustainable Development Goal 3. The specific objectives are to support Member States to prepare for and prevent disease outbreaks and health emergencies; promptly detect, speedily report, and confirm outbreaks; strengthen and sustain capacity to promptly respond to and recover from the negative effects of outbreaks and health emergencies. To achieve these objectives, each flagship programme proposed a set of activities and plans that are aligned to the 5-year goal of rolling out all three flagships to the entire African continent. For PROSE, the goal for 2022 was to determine a clear roadmap with each of the 17 countries and begin implementation of activities. For TASS the goal was to assess the needs of the targeted countries, define implementation modalities and provide laboratory strengthening services. And for SURGE, the goal was to make sure countries have the workforce, operations, and logistics support, and the coordination mechanism needed to stop the next pandemic. An initial set of 17 countries were targeted for the roll out in 2022 with the intent to scale up to the whole of the continent. To this end, there is the need to answer some broad questions, (i) How effective are the flagships; (ii) What combinations of flagship interventions have a maximum impact on preparedness, detection, response, and health systems resilience—the game changers; (iii) What enabling environment required for implementation of flagships; and (iv) What is missing (that could have more impact).

In line with WHO’s Core Function of Leadership and support for research in health EPR, and Transformation focus area of Strengthening capacity in use of evidence for health policy and action, the process of setting the agenda for EPR research in Africa was initiated with three broad areas of focus namely implementation, clinical and epidemiological. This would ensure that there are clear research priorities that would contribute to strengthening EPR capacities in the region.

The research priority setting was implemented through a systematic process of a consensus building. The methodology of the Child Health and Nutrition Research Initiative [12] was used in the prioritization exercise. This systematic process of research priority setting comprises 15 steps that begin with the selection of stakeholders/participants and span through choosing a limited set of the most useful and important criteria, listing of a large number of proposed health research options, scoring of the health research options using the chosen set of criteria, and calculating overall priority scores and assigning ranks. This methodology has been used extensively in research prioritization exercises with demonstrated practicality at institutional, regional, national, international, or global levels, and it has a general appeal among policy makers, development partners and researchers, and it supports the participation of a wide range of stakeholders [13, 14].

**Fig. 1:**
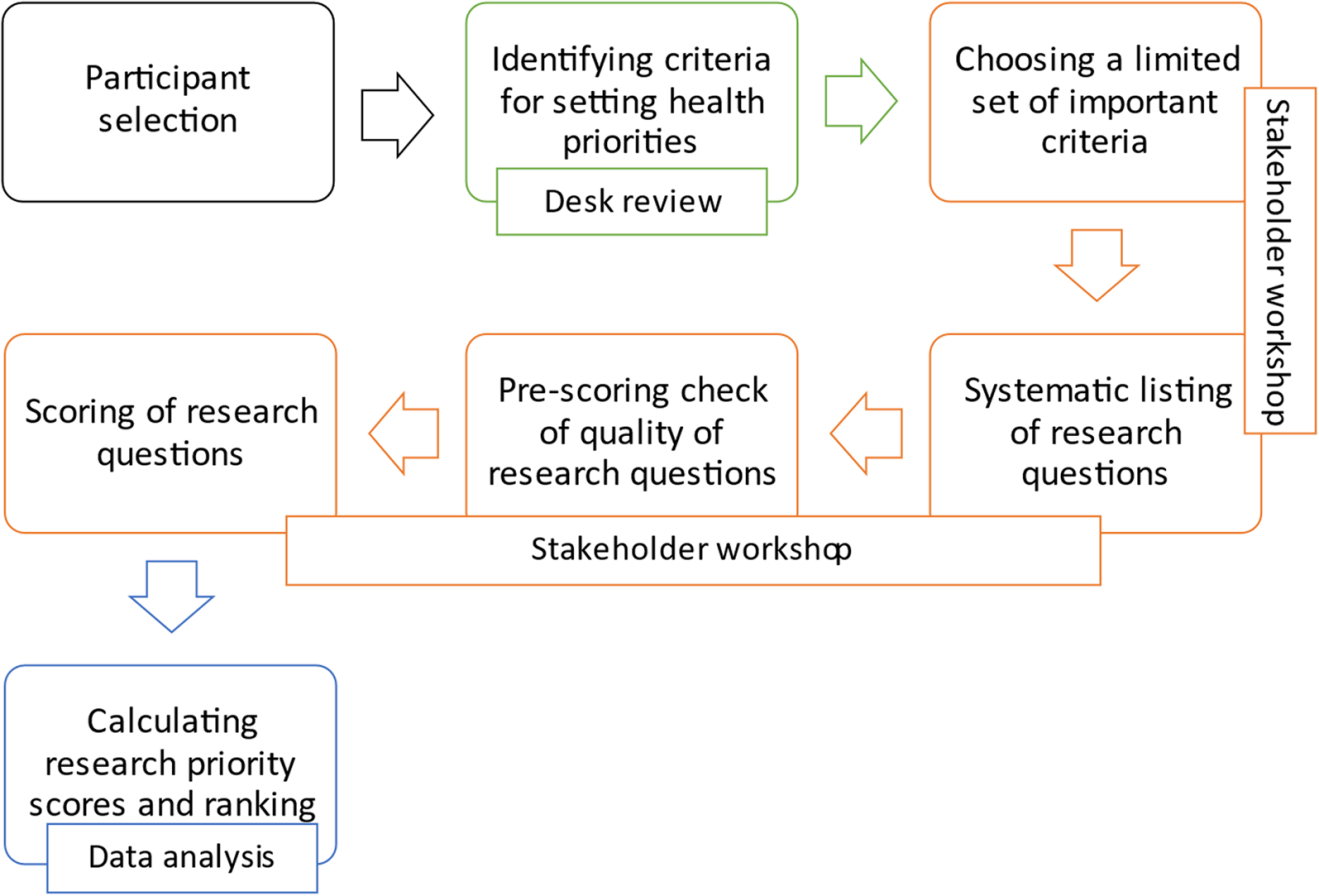
Flow chart of the methodological process of research priority setting for EPR in Africa

The specific methods that were used in the priority setting were desk review and a stakeholder workshop. The purpose of the desk review was to identify various criteria that have been used to set research priorities and how these criteria were operationalized (defined and applied). The aims of the stakeholder workshop were to identify issues in EPR to be addressed through research, and to select the research priorities based on an agreed set of assessment criteria. The entire process from participant selection to data analysis is highlighted in Fig. 1.

#### Desk review

A rapid review of documents was conducted. The search algorithm included various combinations of the following key terms: criteria, research priority, research prioritization, research agenda, public health emergency, preparedness, response. Searches were performed on Google for relevant documents including articles, reports of expert meetings, official/public documents, and programme reports. Minutes and reports of previous research priority setting meetings were also retrieved through email exchange from experts.

The criteria for assessment of research questions were compiled with a working definition for each criterion. The starting list was adapted from relevant documents (articles and reports) on research priority setting including the guidelines that were developed by CHNRI for priority setting in health research investment and the WHO global research agenda for family planning [12, 15, 16]. Both activities involved systematic processes of ranking and consensus-building among experts. The need for a systematic approach to the specification of the research prioritization criteria is well documented in literature [12, 17–20]. Table 1 shows the names and definitions of the assessment criteria.

**Table 1:** Names and definitions of criteria for research prioritization

Priority setting criteria	Definitions
Affordability	Research is expensive; the answering of some research questions may be more or less affordable within the context
Answerability	Is the research question answerable in the given context?
Attractiveness	Appeal to stakeholders/end users. Attractive to a wider range of stakeholders
Equity	Implications for equity (fairness) in access to resources
Feasibility	All other criteria being equal, answering some research questions may be more or less feasible in the real-world setting: issues to consider relate to acceptability of the research question, capacity of end-users to implement the research, appropriateness given the intervention context
Novelty	Likely to generate truly innovative knowledge
Potential for translation	Likely to generate knowledge which is usable beyond the immediate implementation context—also links to impact **(Deliverable at scale)**
Potential to have impact	Impact of research questions on the implementation of the intervention Impact of the answers to research questions on the broader field of implementation studies
Public opinion	Justified (justifiable?) and acceptable to the general public
Involvement of end-users	Possibility of high-quality involvement of end-users of the research
Community involvement	Possibility of high-quality involvement of the target or beneficiary communities
Ethical aspects	Unlikely to raise ethical concerns
Cost-effective	Research is likely to generate knowledge that is valuable (relevant, novel, useful, etc.) for money spent
General public health benefits	Research is likely to generate knowledge that is useful for disease prevention and improving the health of people and their communities

#### Stakeholder consultation workshop

The stakeholder consultation workshop was a five-day hybrid event that lasted from 17 to 21 October 2022. Online participants joined the workshop through Zoom. Unique meeting invite links were sent to registered participants. To ensure meaningful participation of online participants, the virtual meeting room was unmuted during the workshop and each in-person attendee had access to a micro-phone and headset. Remote participants were intentionally and personally invited to speak or comment during the workshop, and questions or comments posted in the chat were read out by the facilitators. To give remote participants a greater presence in the room, two monitors were set up in the room and the main screen was used to project life size images of remote participants.

Three stages of consensus building were conducted: (i) Structured brainstorming to select the criteria for ranking of research questions/topics and a list of research questions based on identified needs; (ii) Two-round modified Delphi exercise to establish consensus on the most relevant/suitable criteria for assessing the research questions; and (iii) Two-round modified Delphi exercise to establish consensus on research priorities. The steps are discussed in detail in the next paragraphs.

A total of 29 experts participated in the workshop, which was held in Brazzaville, Congo (Table 2). The workshop participants were technical focal points from the different programme areas in EPR and the Assistant Regional Director Cluster of WHO AFRO. Others were EPR focal points from country offices, representatives of WHO EPR hubs in Dakar and Nairobi, representatives from five ministries of Health (Nigeria, Senegal, Democratic Republic of Congo, Kenya, and Ethiopia), representatives from Africa Center for Disease Control, the WHO Headquarters in New York, United Nation’s Children Fund and from research institutions in SSA. The activities that were undertaken in each of the three stages of consensus building are presented in the ensuing sub-sections.

**Table 2:** Characteristics of participants

ID	Gender	Organization/Sector	Country
1.	Male	Africa Centre for Disease Control	Ethiopia
2.	Male	WHO RO	Kenya
3.	Male	Director Public Health (Ministry of Health)	DRC
4.	Male	Director Public Health (Ministry of Health)	Nigeria
5.	Male	EPHI-Ministry of Health	Ethiopia
6.	Female	WHO—EPR Research Manager	DRC
7.	Female	WHO—EPR Research Officer	Nigeria
8.	Female	EPR/WHO	Ethiopia
9.	Male	EPR/WHO	HQ—New York
10.	Male	EPR/WHO	AFRO-HQ
11.	Male	University of Nigeria/Academic	Nigeria
12.	Male	IHM	DRC
13.	Male	WHO—Lead IT	DRC
14.	Male	Ministry of Health	Senegal
15.	Female	Ministry of Health	Gabon
16.	Male	Nigeria Centre for Disease Control	Nigeria
17.	Female	WHO—Public Health Officer	South Africa
18.	Female	Science for Africa/Research	Kenya
19.	Female	University of Nigeria/Academic	Nigeria
20.	Male	VPD/WHO	Congo
21.	Male	WHO Health Emergency Programme (WHE)	HQ—New York
22.	Female	WHO Research officer	Zimbabwe
23.	Male	WHO Research officer	Ethiopia
24.	Female	WHO Research officer	Congo
25.	Male	WHO Research officer	Kenya
26.	Male	WHO Research officer	Gabon
27.	Male	WHO/AFRO—EPR	DRC
28.	Male	WHO/AFRO—EPR	DRC
29.	Male	WHO/AFRO—EPR	DRC

##### Structured brainstorming

Structured brainstorming is a systematic process which encourages active participation in contributing ideas towards a specific goal, in a non-critical or non-evaluative environment. Participants are given a fair chance to voice their ideas such that the discussion is not dominated by one person or a few people. In this workshop, structured brainstorming was used to: (i) identify (from experience or knowledge) the challenges and knowledge gaps in EPR in SSA; and (ii) generate a starting list of research questions that can contribute to addressing the challenges and gaps in the three broad research areas of the EPR.

**Group work:** The brainstorming exercise began with a random assignment of participants into four groups. Each group was asked to reflect on the three broad areas of the EPR programme in SSA, and identify from their experiences and/or knowledge, (i) the challenges (problems, capacity needs, etc.) that the programme faces in each broad areas; and (ii) the knowledge (research and development) gaps that need to be filled—to better understand current and future challenges, effective interventions for strengthening country EPR capacities, and potential strategies for optimizing the effectiveness of interventions.

Having generated ideas on challenges and knowledge gaps, each group was asked to articulate specific research questions (or topics) that will generate answers or solutions to the challenges identified and contribute to filling the gaps in knowledge in EPR in the region. The ideas were recorded in a uniform Microsoft Word template with comprising four broad areas—Epidemiology, Clinical, programme Implementation and Cross-cutting.

**Plenary discussion:** Outputs from each group were projected and presented for critical review and feedback from facilitators and participants, and for discussion in plenary. Groups received comments and suggestions for refining their ideas and research questions. Revised outputs from each group were submitted and circulated to all the participants for collation and synthesis.

**Thematic collation and synthesis of ideas:** Participants were reassigned to three groups—Epidemiology, Clinical and Implementation—based on their expertise, interest and/or area of work. Each group was tasked with the responsibility to collate (assemble) all challenges, knowledge gaps and research questions that are recorded in the templates for their assigned broad area (theme), and to removing any duplicates. They were additionally requested to record any cross-cutting issues and research questions that were not captured on the lists.

**Generation of consolidated list of research questions:** An initial list of research questions was generated by merging the updated list of research questions from each broad area. Excluding duplicates, all research questions that were generated and adopted/adapted by participants were retained.

##### Prioritization of assessment criteria using the Delphi technique

The Delphi technique is a consensus-building method of eliciting and refining judgements from a group of people, in order to generate knowledge that is currently not available [21]. The three main features of the technique—anonymous response, iteration and controlled feedback, and statistical group response—are designed to minimize the influence of dominant individuals in group interactions, and the biasing effects of irrelevant communication, and group pressure towards conformity. In this workshop, modified Delphi technique was used to achieve the consensus opinion of participants on, (i) the set of criteria to be used to assess research questions, and (ii) the research priorities in EPR for SSA.

Although there are several criteria that can be used to define health research priorities, stakeholders should carefully select the most suitable ones for the context of prioritization[17, 22, 23]. The key considerations in the study were the programme context of EPR in SSA, to ensure that the research priorities were aligned to the needs of the programme and the values of the key stakeholders in the programme.

Two rounds of Delphi exercise were undertaken to establish consensus on the most relevant/suitable criteria for assessing research questions. In round one, the starting list of 14 criteria was presented and participants were asked to rank the criteria from the most relevant or suitable to the least relevant or suitable for assessing research questions in EPR. The first round of ranking was followed by plenary discussions on the rationale for ranking the criteria.

In the second round of the modified Delphi, participants got a second chance to re-rank the 14 criteria in order of relevance or suitability. The first and second ranking exercises were through live online polling. Seventeen [12] participants voted in the first-round while 29 voted in the second round.

##### Prioritization of research questions

In the first round of the modified Delphi, the initial list of research questions was shown to participants (in thematic groups) and they were asked to vote to keep, remove or modify the research questions based on their assessment of the quality and relevance of the question to EPR capacities in the region. The voting was through live online polling. Research questions were ranked high (to keep), medium (to modify) and low (to remove). Consensus was set a priori at 50% agreement with any of the available options. Percent agreement is the basis for definition of consensus for many studies that adopt the Delphi method [24]. The decision for 50% consensus was made based on the suggestions of participants and the agreement that this was an acceptable cut-off for deciding which research questions should be kept, modified, or dropped before the ranking exercise.

In the second round of the modified Delphi, the condensed list of research questions was coded on the Open Data Kit software and each research question was ranked from 1 to 5 in each of the selected assessment criterion. Table 3 shows the ranking/scoring template. *(A rank of 1 meant that for that criterion the research question was considered to rank very low; a score of 2 signified low ranking; a score of 3 signified moderate ranking; 4 signified high ranking; and 5 signified very high ranking)*. Scores assigned to each question were tallied, by criterion and the question with the highest score was given the highest priority. Research priority score (RPS) was calculated for each research question as the mean of the criteria scores for 29 participants. The maximum achievable RPS per question is 145 (5*29) and the minimum is 29 (1*29).

**Table 3:** Ranking/scoring template for first ranking exercise

Research question	Criteria							Total score
	C1	C2	C3	C4	C5	C6	C7	
Q1								
Q2								
Q3								
Q4								
Q5								
Q…123								

## Results

### Ranking of assessment criteria

**Fig. 2:**
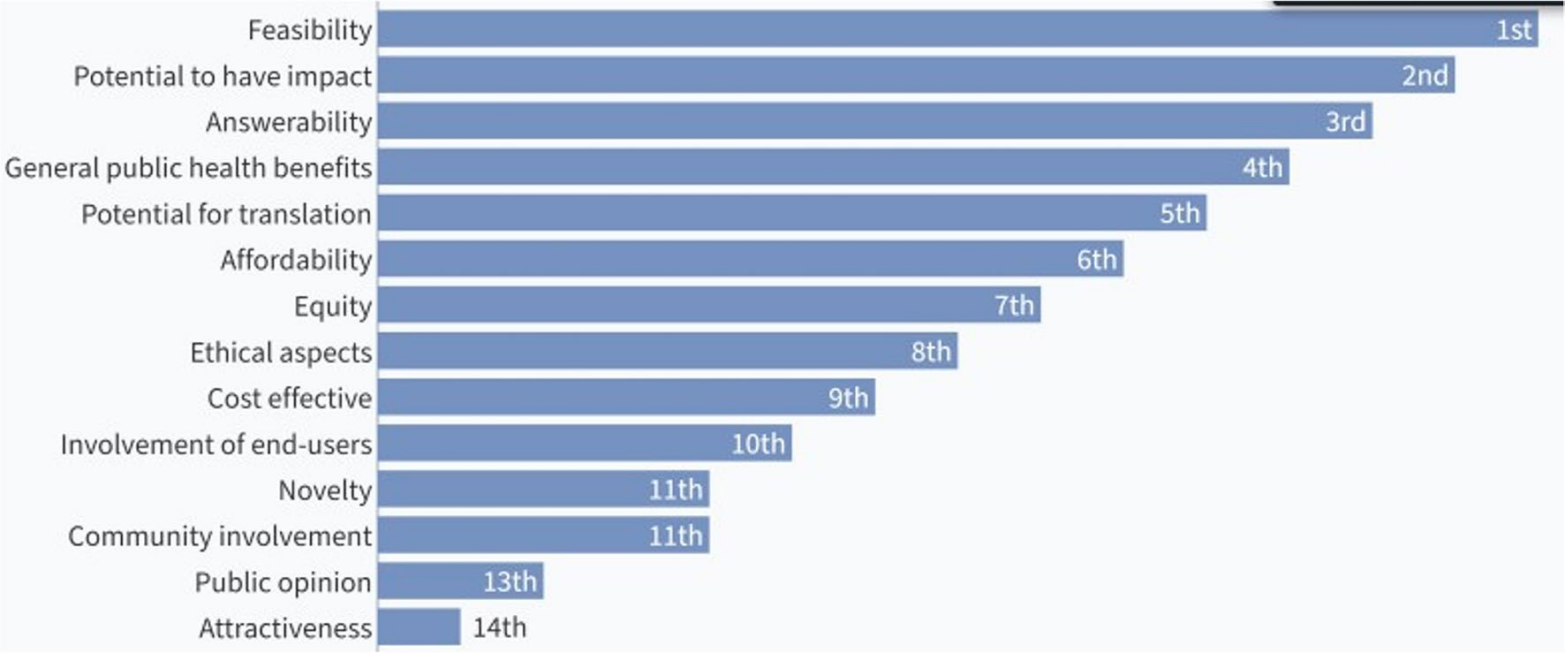
Results of the first ranking of criteria for assessing research questions

**Fig. 3:**
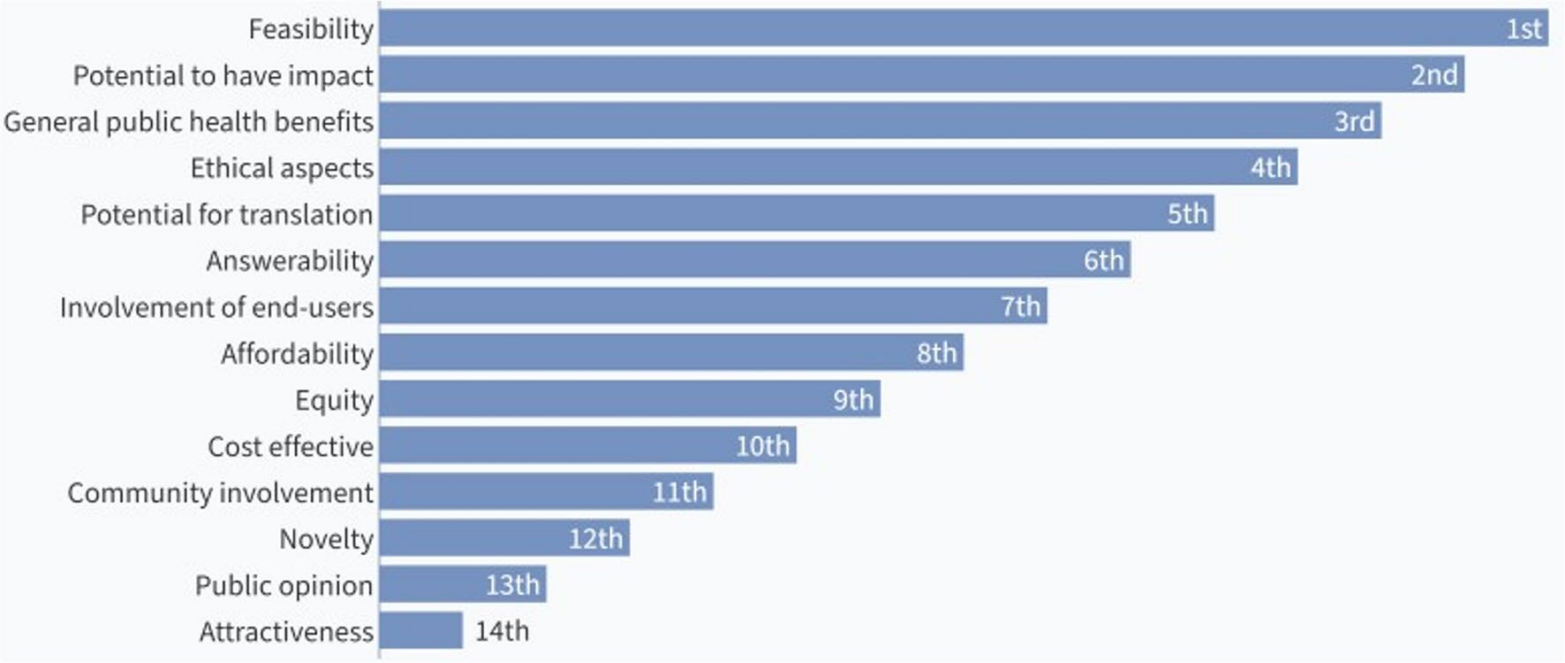
Results of the re-ranking of criteria for assessing research questions

The results of the ranking and re-ranking of the assessment criteria are shown in Figs. 2 and 3, respectively. The top seven ranked criteria from the first round were feasibility, potential to have impact, answerability, public health benefits, potential for translation, affordability, and equity. After the second round of voting, feasibility, and potential to have impact retained their positions as the top two ranked criteria. Potential for translation also retained its position as the fifth criteria. General public health benefits moved up to the third ranked criteria while answerability moved down to 6^th^ position. Affordability and equity moved down to the bottom half of the ranks while ethical aspects and involvement of end-users occupied the 4^th^ and 7^th^ positions, respectively.

Participants decided that since potential to have impact encompasses public health benefits, the latter should be considered a part of the former, and affordability should be included in the list of seven criteria that will be used to assess the research questions.

### Generation and ranking of priority research questions.

A total of 123 research questions were generated. This comprised 29 questions on the Epidemiology broad area, 22 on the Clinical area, 23 on Implementation, and 49 on cross-cutting issues. The highest ranked question achieved a research priority score of 123.0 while the least ranked question had a score of 89.0. The condensed list of priority research questions with their corresponding RPS is attached as a supplementary file [see Additional file 1]. Also attached as a supplementary file is the disaggregated list of the research questions by the EPR broad areas [see Additional file 2].

Table 4 shows the research priority scores of the top 25 research questions and the corresponding broad areas. Nine of the questions were on the clinical broad area, and eight each were on the Epidemiology and Implementation broad areas. None of the top 25 questions was on a cross-cutting issue.

**Table 4:** RPS for the research questions/topics for strengthening EPR capacities in Africa region

Broad EPR area	Research questions	Research priority score
Clinical	What are the knowledge and skills gaps among healthcare workers in detecting and responding effectively to disease outbreaks?	123.0
Epidemiology	What are the factors affecting the quality of data (accuracy, timeliness, completeness) for epidemic prone diseases?	121.3
Clinical	What are the factors responsible for vaccine hesitancy?	121.1
Clinical	What factors lead to increased transmission of infections among health care workers during public health emergencies? What measures are effective for ensuring that health workers are protected from highly infectious pathogens in public health emergencies?	120.4
Epidemiology	What are the best strategies to improve the effectiveness of contact tracing for epidemic prone diseases?	120.3
Clinical	Are the current case definitions effective as screening tools for epidemic and pandemic prone diseases?	119.0
Epidemiology	How can national and sub-national laboratory capacity for timely disease confirmation be strengthened within the Integrated Diseases Surveillance and Response framework?	118.9
Epidemiology	What are the factors affecting prompt data sharing on epidemic-prone diseases?	118.3
Implementation	What strategies are effective for appropriate community participation in EPR?	117.7
Clinical	What training modalities build lasting capacity and improved performance for health emergency preparedness and response? Why are these successful and how can they be scaled up and sustained?	117.6
Epidemiology	How can research results be best applied to ensure a more effective and rapid response across all scales of emergencies?	117.1
Clinical	What measures are effective for improving the quality of care provided to patients in the treatment centers for highly infectious pathogens?	116.4
Epidemiology	What are the minimum requirements for timely response to disease outbreaks?	115.7
Implementation	What are the factors affecting health workers retention?	115.7
Clinical	What are the barriers and how can we strengthen mechanisms for vaccines and pharmaceuticals trials for infectious diseases in Africa?	115.6
Implementation	What are the factors responsible for poor compliance of community for emergency health intervention?	115.1
Implementation	What is the appropriate skill mixed for effective EPR?	114.6
Clinical	How can we better leverage innovations and technology to capacitate health services and facilities in under-resourced locations to improve health outcomes through better clinical characterization?	114.4
Implementation	What are the challenges and enablers for community engagement?	114.4
Epidemiology	What are the factors responsible for uptake of preventive interventions for seasonal diseased outbreaks?	114.3
Epidemiology	How can community active case finding for epidemic prone diseases be established and implemented?	114.1
Clinical	How can we get simplified technologies available and accessible at the points of care in underserved rural areas to improve case management of comorbidities in the context of emergencies?	113.9
Implementation	How do risk communication mechanisms empower community engagement?	113.6
Implementation	How to we build a data architecture for EPR within the African context?—data architecture triangulates data from different sectors	113.6
Implementation	What are the factors that affect the use of evidence in planning and decision making?	113.4

The top ten research priorities were on issues around the knowledge and skills of healthcare workers in detecting and responding effectively to disease outbreaks; quality of data (accuracy, timeliness, completeness) for epidemic prone diseases; determinants of vaccine hesitancy; determinants of infection transmission among health care workers during PHE; effective measures for protecting health workers from highly infectious pathogens in PHE; strategies to improve the effectiveness of contact tracing for epidemic prone diseases; effectiveness of current case definitions as screening tools for epidemic and pandemic prone diseases; measures to strengthen national and sub-national laboratory capacity for timely disease confirmation within the Integrated Diseases Surveillance and Response framework; factors affecting prompt data sharing on epidemic-prone diseases; and effective strategies for appropriate community participation in EPR. Other top research priorities are as shown in Table 4.

Table 5 shows the disaggregated and total proportions of research questions that achieved ≥ 75% (high), 50–75% (medium) or < 50% (low) research priority scores out of the maximum achievable research priority score (which is 145).

**Table 5:** Level of research priority scores of the disaggregated by the EPR broad areas

Broad areas of EPR	N	Levels (range) of research priority scores n (%)		
		High (RPS = 108.75–145)	Medium (RPS = 72.5–108.74)	Low (RPS < 72.5)
Clinical	22	16 (72.73)	6 (27.27)	0
Epidemiology	29	14 (42.28)	15 (51.72)	0
Implementation	23	14 (60.87)	9 (39.13)	0
Cross-cutting	49	8 (16.33)	41 (83.67)	0
Total	123	52 (42.28)	71 (57.72)	0

A total of 52 (42.28%) research questions had research priority scores that were at least 75% of the maximum achievable score. Most of the research questions on the clinical broad area (72.73%) and the implementation broad area (60.87%) had high research priority scores. Whereas 42.28% of the questions on epidemiology had high research priority scores. Only 16.33% of the cross-cutting research questions achieved high research priority scores. None of the research questions had a research priority score that was less than 50% of the maximum achievable score. This implies that all the research questions were judged to be of above average priority to the stakeholders.

## Discussion

Our findings show that within the context of WHO AFRO’s Emergency Preparedness Programme, stakeholders generated a long list of research questions and topics that they adjudged to be relevant for strengthening capacities for EPR within the African region. However, to provide valuable direction for the allocation of public and private research funds, the stakeholders had to decide, through consensus-building, which research questions were more or less important.

Our study shows that consulting with a diverse group of stakeholders ensures that differing and distinct views and perceptions are brought to the fore. The importance of stakeholder engagement in setting research priorities as also reported elsewhere [12, 23]. It is important that the key stakeholders are involved in the research priority setting and that their interests are taken into consideration in the prioritization process. However, the difficult task of managing the often-conflicting interests of stakeholders is acknowledged, particularly when there is uneven distribution of power among the stakeholders. Hence, in setting the EPR research priorities, the facilitators employed a mix of strategies for effective stakeholder engagement, including, the purposive selection of participants, and consensus-building through iterations of group creativity activities and individual ranking exercises.

The process of determining the strategic importance of research questions could be tedious for stakeholders if there are no clear criteria for assessment. The specification of criteria enables a more rational process of priority setting, particularly where the research questions are brand new [20, 22]. In our research priority setting exercise, the use of a systematic and transparent approach of consensus-building to define the set of criteria for assessing the newly formed EPR research questions was a plausible approach to ensure widespread consensus and ownership of the final research priorities by all the participants. Adopting a methodologically transparent approach in consensus-building aligns with existing literature on stakeholder management [25]. Methodological transparency is closely tied to trust and credibility in consensus-building processes [25]. Stakeholders are more likely to trust the outcomes of a consensus-building effort when they can clearly understand and evaluate the methods used and can see how decisions are made.

The top twenty-five research questions that were prioritized for strengthening EPR capacities in the African region align with the global capacity needs for effective EPR [26, 27]. Other research priorities that resonate with exiting literature include understanding the determinants of vaccine hesitancy, strengthening community participation in EPR, protection of health workers from highly infectious pathogens during PHE, and strengthening national and sub-national laboratory capacity for timely disease confirmation.

These research priorities address some of the demand- and supply-side barriers to effective EPR in the African region, the knowledge gaps in EPR, and some recommended strategies for effective management of PHE [28–32]. Although the Emergency Preparedness Programme of WHO AFRO aims to strengthen country capacities in emergency preparedness and response across the three broad areas of the Programme, attention should also be paid to fundamental health systems issues that underline overall health system resilience and responsiveness during public health emergencies.

The major limitations of research priority setting are, (i) the likelihood of excluding some key stakeholders in the prioritization process and missing some relevant research questions; (ii) the potential influence of dominant individuals; (iii) the biasing effects of irrelevant reflections from dominant speakers; and (iv) the potential to conform to the popular opinion. In this prioritization exercise, stakeholders at the frontlines of EPR in the AFRO member states were not included in the exercise. The EPR office of WHO AFRO is planning some webinar sessions to get additional inputs on research priorities from more stakeholders and all member states. A face-to-face validation workshop is also being planned and this will engage a wider range of stakeholders. The use of modified Delphi technique minimized the effects of the other limitations and ensured that each participant’s opinion contributed to the final research priorities.

## Conclusion

The stakeholder consultation workshop was successful in developing a starting set of research priorities for EPR in Africa. The prioritization exercise led to the selection of the most useful research questions for strengthening EPR capacities in SSA. The next steps would be to get the WHO member countries in the region to buy into these research priorities, and to subsequently develop context-specific research agenda, implementation frameworks and operational guidelines. Action needs to be taken to continuously update the research agenda so that the research priorities are time and context sensitive.

### Supplementary Information

**Additional file 1. **Comprehensive list of research questions and topics and corresponding Research Priority Scores in descending order of ranking

**Additional file 2.** Disaggregated list of research questions and topics by EPR broad areas, and corresponding Research Priority Scores

## Data Availability

All data generated or analysed during this study are included in this published article [and its supplementary information files].

## Abbreviations

AFRO: African Regional Office
EPR: Emergency preparedness and response
PHE: Public health events/emergencies
SSA: Sub-Saharan Africa
