# Supplementary material for: What are the research priorities for strengthening public health emergency preparedness and response in Africa?
Source: Health Res Policy Syst. 2023 Oct 23;21:107. doi: 10.1186/s12961-023-01059-6 (PMC10594758; doi:10.1186/s12961-023-01059-6)
Supplement: Supplementary file 2 — Additional file 2. Disaggregated list of research questions and topics by EPR broad areas, and corresponding Research Priority Scores [file 12961_2023_1059_MOESM2_ESM.docx]

## **Additional file 2. Disaggregated list of research questions and topics by EPR broad areas, and corresponding RPS in descending order of ranking**

**Epidemiology broad area for EPR in Africa region**

| **Research Questions in Epidemiology (n = 29)** | **RPS** |
| --- | --- |
| What are the factors affecting the quality of data (accuracy, timeliness, completeness) for epidemic prone diseases? | **121.3** |
| What are the best strategies to improve the effectiveness of contact tracing for epidemic prone diseases? | **120.3** |
| How can national and sub-national laboratory capacity for timely disease confirmation be strengthened within the Integrated Diseases Surveillance and Response framework? | **118.9** |
| What are the factors affecting prompt data sharing on epidemic-prone diseases? | **118.3** |
| How can research results be best applied to ensure a more effective and rapid response across all scales of emergencies? | **117.1** |
| What are the minimum requirements for timely response to disease outbreaks? | **115.7** |
| What are the factors responsible for uptake of preventive interventions for seasonal diseased outbreaks? | **114.3** |
| How can community active case finding for epidemic prone diseases be established and implemented? | **114.1** |
| How does surveillance impact on effective response to health emergencies? | **113.0** |
| How can genomic surveillance be improved in countries for effective response to health emergencies? | **113.0** |
| How can countries be supported to undertake genomic surveillance as part of the Integrated Diseases Surveillance and Response? | **112.9** |
| How can disaster risk management be strengthened in the African setting? | **112.0** |
| How can information technology and innovation improve contact tracing for epidemic prone diseases? | **111.3** |
| What are the socio-ecological factors affecting disease outbreaks in Africa? | **109.9** |
| What are the factors associated with the delay in detecting zoonotic diseases outbreaks? | **108.1** |
| How can outbreaks in animals be promptly detected and reported for One Health response? | **107.9** |
| How can the national surveillance system be more resilient in the context of a humanitarian crisis? | **106.3** |
| Which Key Performance Indicators and tool will adequately support the assessment of contact tracing activities in health emergencies? | **106.1** |
| What are the most appropriate methods to strengthen capacity for modelling and research in emergency preparedness and response? | **106.1** |
| What are the drivers for spread of pathogens from animals to humans? | **106.0** |
| How can appropriate modelling be used for better decision making? | **104.6** |
| What are the best criteria to select the most effective tools for the accurate prediction of known epidemic prone diseases? | **103.1** |
| What are the geographic and temporal patterns of pathogens in the region? | **103.0** |
| How can predictive modelling be used to understand the impact and severity of diseases? | **102.4** |
| What are the reservoirs of major pathogens of zoonotic diseases? | **102.0** |
| What are the transmission dynamics of infectious diseases in different African contexts? | **101.7** |
| How do environmental changes in Africa impact the ecology of zoonotic diseases and how can we monitor these trends? | **101.0** |
| How can simulation scenarios be developed to better predict epidemic-prone diseases in Africa? | **98.1** |
| What is the susceptibility of different at-risk population groups to different diseases? | **93.1** |

**Clinical broad area for EPR in Africa region**

| **Research Questions in Clinical broad area (n = 22)** | **RPS** |
| --- | --- |
| What are the knowledge and skills gaps among healthcare workers in detecting and responding effectively to disease outbreaks? | **123.0** |
| What are the factors responsible for vaccine hesitancy? | **121.1** |
| What factors lead to increased transmission of infections among health care workers during public health emergencies? What measures are effective for ensuring that health workers are protected from highly infectious pathogens in public health emergencies? | **120.4** |
| Are the current case definitions effective as screening tools for epidemic and pandemic prone diseases? | **119.0** |
| What training modalities build lasting capacity and improved performance for health emergency preparedness and response? Why are these successful and how can they be scaled up and sustained? | **117.6** |
| What measures are effective for improving the quality of care provided to patients in treatment centres for highly infectious pathogens? | **116.4** |
| What are the barriers and how can we strengthen mechanisms for vaccines and pharmaceuticals trials for infectious diseases in Africa? | **115.6** |
| How can we better leverage innovations and technology to capacitate health services and facilities in under-resourced locations to improve health outcomes through better clinical characterization? | **114.4** |
| How can we get simplified technologies available and accessible at the points of care in underserved rural areas to improve case management of comorbidities in the context of emergencies? | **113.9** |
| How can we strengthen the capacity for standard bureaux in Africa to certify and validate diagnostic kits for emergencies? | **112.3** |
| What is the model for country and regional mechanisms to accelerate product development and local manufacturing for medical countermeasures in Africa? | **110.3** |
| How can information technology innovations strengthen emergency response systems by improving situational awareness, data sharing and decision support for the public health workforce? | **110.0** |
| What are the social constructs that influence disease outbreaks and health seeking behaviour? | **109.9** |
| How do we measure the sustainable impact of timing of the training on the response to a public health emergency? | **109.9** |
| How long does immunity to a given infectious disease last after vaccination? | **109.9** |
| What are the effective simplified approaches to management of acute malnutrition in fragile, conflict and vulnerable (FCV) settings? | **109.1** |
| How can the health system quickly adopt electronic medical record systems in health emergencies? | **108.0** |
| What is the status of and access to specialized care in the African context for emergency response and how do referral systems contribute to improved access to this care? | **106.7** |
| How can we harness international industrial capacities to develop therapeutics for the management of prevalent and novel pathogens including Lassa fever in Africa? | **106.0** |
| What are the characteristics of transmission of infectious pathogens between outbreaks? | **105.1** |
| Why do some infectious pathogens persist in some geographical regions and population groups? | **102.1** |
| What was the coverage of telehealth and remote medicine during the COVID-19 pandemic? | **101.9** |

**Implementation broad area of EPR**

| **Research Questions in Implementation (n = 23)** | **RPS** |
| --- | --- |
| What strategies are effective for appropriate community participation in EPR? | **117.7** |
| What are the factors affecting health workers retention? | **115.7** |
| What are the factors responsible for poor compliance of community for emergency health intervention? | **115.1** |
| What is the appropriate skill mixed for effective EPR? | **114.6** |
| What are the challenges and enablers for community engagement? | **114.4** |
| How do risk communication mechanisms empower community engagement? | **113.6** |
| How to we build a data architecture for EPR within the African context? - data architecture triangulates data from different sectors | **113.6** |
| What are the factors that affect the use of evidence in planning and decision making? | **113.4** |
| What are the barriers and enablers for uptake of recommendations from research and reviews? | **113.3** |
| What metrics are available and effective for assessing public health system preparedness and response at district or local government levels? | **113.1** |
| How can research products (be) more available to inform policy and practice? | **113.0** |
| How do we strengthen the data architecture to capture population and heath information for emergency preparedness and response? | **111.7** |
| What are the enablers and barriers responsible for poor compliance of community for emergency health intervention? | **111.1** |
| What methods (including the study of real incidents) are effective for informing benchmarks that can be built into a continuous quality improvement system for public health preparedness? | **109.3** |
| Are the current preservice curricula adequate for the current EPR in African countries? | **108.0** |
| What are the strategies to build the leadership capacity at the different levels for EPR? | **107.7** |
| What are the best ways to make research accessible to target audience? | **107.6** |
| How can we address the factors responsible for poor compliance of community for emergency health intervention? | **106.7** |
| How do we sustain successful initiatives in knowledge translation? | **106.7** |
| How can we build the country capacity to make a case for investing in EPR? | **106.6** |
| Why are we not implementing enablers for community engagement? | **103.6** |
| How do we spur African countries to prioritise EPR? | **103.1** |
| Why don’t we implement known facilitating factors to knowledge translation? | **97.0** |

**Cross-cutting research priorities in EPR**

| **Cross-cutting Research Questions in EPR (n = 49)** | **RPS** |
| --- | --- |
| What are the enablers and barriers to manufacturing emergency preparedness and response commodities (personal protective equipment (PPE), pharmaceuticals and diagnostics) in the region? | **113.0** |
| What are the criteria and metrics for effective risk communication in emergency situations with (1) the public health workforce, (2) emergency response partners, (3) the media, (4) the public, and (5) vulnerable populations? | **112.0** |
| How can local teams be trained and empowered to deliver healthcare in conflict and humanitarian settings in Africa? | **111.6** |
| What innovative approaches are being used to deliver healthcare to populations in conflict and humanitarian settings, and how can these be adapted in other conflict settings in Africa? | **111.3** |
| What are the factors affecting effective operationalization of incident management systems (IMS) for health emergencies at the country level? | **111.0** |
| What strategies mechanisms can support effective institutionalization of multisectoral response during health emergencies? | **109.6** |
| How can we use the lessons learnt from the field epidemiology training to improve the competency-based training for other responders (health and non-health) in EPR? | **109.1** |
| What are the factors that generate (enable create) sustainable demand for local innovative solutions (to improve the management of health crises) in the African region? | **108.9** |
| How can new technologies (e.g., Internet and web-based technologies, and cellular text messaging) be better used to fill risk communication gaps in emergency settings, including those experience by vulnerable populations? | **108.7** |
| How effective is “just-in-time” training during disasters, and what is the most effective way to prepare ahead of time to deliver it? | **108.3** |
| What is an effective model for stimulating regional collaboration between public private sector and academia to developing countermeasures for diseases that disproportionately affect Africa? | **108.1** |
| How effective is the one health coordination platform in preparing for disease outbreaks? | **107.7** |
| What model statutes or agreements would enhance the private sector’s participation in preparedness efforts? | **106.6** |
| Why did the existing early warning mechanisms fail to prepare for disease outbreaks? | **106.4** |
| How can new technologies be applied to improve the training and education of the public health workforce on EPR? - pre and post service | **106.3** |
| Which risk communication messages motivate people, especially vulnerable populations, to take protective action and engage in appropriate behaviours related to emergencies at different scales? | **106.1** |
| How does data governance influence data sharing for emergency preparedness and response? | **105.7** |
| What is the impact of climate change in the trend of disease outbreak? | **104.7** |
| What are the existing mechanisms for forecasting natural disasters and how can these early warning systems be effectively deployed to trigger quicker response to health emergencies? | **104.7** |
| How can new technologies be applied to improve the training and education of the public health workforce? | **104.6** |
| How do we establish a prevention focused system for early identification of special needs population in advance of and during disaster? | **103.7** |
| What is an effective model to unlock sustainable financing to accelerate generation of new technologies and products? | **103.4** |
| What are the factors that contribute to severity of disease in low resource settings? | **103.3** |
| What are the appropriate criteria for evaluating public health emergency preparedness, response, and recovery? Priority areas include (1) the public health workforce; (2) information management; (3) emergency communications; (4) vulnerable populations; and (5) response systems | **103.3** |
| What are the workforce surge requirements for scenarios varying in duration, type of injury illness, and number of affected individuals? | **102.9** |
| What are the barriers preventing effective translation of pre-emergency and emergency communication strategies to practitioners? | **102.7** |
| How can non-physician medical staff and nonmedical personnel be best utilized (both within and outside their usual scope of work) to improve medical and public health surge capacity during an emergency event? | **102.7** |
| Do the quality and characteristics of public health emergency operation plans improve the quality, timeliness, and effectiveness of an emergency response and, ultimately, health outcomes? | **102.1** |
| How can we adapt existing tools and methods to validate public health system preparedness? | **101.9** |
| What are appropriate strategies for decision-making processes in planning, response, and recovery in EPR? These include criteria for implementation, testing, and improvement of the decision-making process | **101.3** |
| What laws need to be enacted or modified to protect responders and volunteers in emergency response? | **101.3** |
| What types of decision-making systems and processes are most effective for medical decision making in mass- casualty events? | **101.1** |
| What lessons for public health preparedness might be learned from quality and safety initiatives in other sectors (e.g., clinical medicine, aviation, occupational safety)? | **101.0** |
| What systems are necessary to effectively organize, allocate, and utilize unsolicited resources (i.e., volunteers and donations)? | **100.1** |
| How can we use the new opportunities with new communication and health care technology, and how might they affect the public health system over time? | **100.0** |
| What is the correlation between climate change and occurrence of diseases (insert disease here) in the African context? | **99.9** |
| What model statutes or agreements would enhance the private sector’s participation in preparedness efforts? | **99.1** |
| What are the legal frameworks (e.g., human-subjects protections) for studying real-time incidents? | **98.7** |
| How can nonphysician medical staff and nonmedical personnel be best utilized (both within and outside their usual scope of work) to improve medical and public health surge capacity during an emergency event? | **98.4** |
| How can we integrate event-based systems into EPR in Africa? | **98.1** |
| What factors drive variations in planning and coordination across response disciplines, sectors and jurisdictions? | **97.9** |
| What are the characteristics of effective systems-level planning for scalable responses (i.e., those that incrementally increase routine capabilities) and catastrophic responses (i.e., those that require rapid deployment of nonroutine capabilities)? Are there differences in the characteristics of planning for each? | **97.6** |
| How does the return on public health emergency preparedness (PHEP) investments compare with that of other security or preparedness investments? Are there lessons to be learned from other domains related to security and preparedness or from other countries? | **97.6** |
| How does public health system financing (how governmental public health entities are financed or the level of financing) affect nongovernmental investments in preparedness and response? | **95.9** |
| How can the “tipping points” that require abrupt changes to alternative response systems be identified, and how are these alternative systems sustained? | **94.7** |
| How does the return on public health emergency preparedness (PHEP) investments compare with that of other security or preparedness investments or cost of doing nothing? Are there lessons to be learned from other domains related to security and preparedness or from other countries? | **94.3** |
| What level or type of connectivity among responders improves the management of emergencies? | **90.4** |
| Can economic incentives be developed or market forces stimulated to encourage preparedness? Do different groups of individuals and organizations respond to different types of incentives? | **90.1** |
| Can monetary incentives, accreditation, or other inducements be used to encourage quality improvement? | **89.0** |
